# Colony-forming unit cell (CFU-C) assays at diagnosis: CFU-G/M cluster predicts overall survival in myelodysplastic syndrome patients independently of IPSS-R

**DOI:** 10.18632/oncotarget.12105

**Published:** 2016-09-18

**Authors:** Bing Li, Jinqin Liu, Shiqiang Qu, Robert Peter Gale, Zhen Song, Ruixian Xing, Junxia Liu, Yansong Ren, Zefeng Xu, Tiejun Qin, Yue Zhang, Liwei Fang, Hongli Zhang, Lijuan Pan, Naibo Hu, Wenyu Cai, Peihong Zhang, Gang Huang, Zhijian Xiao

**Affiliations:** ^1^ MDS and MPN Centre, Institute of Hematology and Blood Diseases Hospital, Chinese Academy of Medical Sciences & Peking Union Medical College, Tianjin, China; ^2^ State Key Laboratory of Experimental Hematology, Institute of Hematology and Blood Diseases Hospital, Chinese Academy of Medical Sciences & Peking Union Medical College, Tianjin, China; ^3^ Department of Medicine, Haematology Section, Division of Experimental Medicine, Imperial College, London, United Kingdom; ^4^ Medical Service Division, Institute of Hematology and Blood Diseases Hospital, Chinese Academy of Medical Sciences & Peking Union Medical College, Tianjin, China; ^5^ Cell culture laboratory, Institute of Hematology and Blood Diseases Hospital, Chinese Academy of Medical Sciences & Peking Union Medical College, Tianjin, China; ^6^ Department of Pathology, Institute of Hematology and Blood Diseases Hospital, Chinese Academy of Medical Sciences & Peking Union Medical College, Tianjin, China; ^7^ Divisions of Experimental Hematology and Cancer Biology, Cincinnati Children's Hospital Medical Center, Cincinnati, Ohio, USA

**Keywords:** myelodysplastic syndromes, colony-forming unit cell, prognosis

## Abstract

**Background:**

*In vitro* colony-forming unit cell (CFU-C) assays are usually-used to detect the quantitative and qualitative features of haematopoietic stem cells (HSCs). We studies CFU-C assays in bone marrow samples from 365 consecutive subjects with newly-diagnosed myelodysplastic syndrome (MDS). Data were interrogated for associations with prognosis.

**Methods:**

CFU-C assays were performed according to the protocol of MethoCultTM H4435 Enriched. 365 consecutive newly-diagnosed, untreated subjects with MDS diagnosed from July, 2007 to April, 2014 were studied. All subjects were reclassified according to the 2008 WHO criteria. Subjects were observed for survival until July 31, 2015. Follow-up data were available for 289 (80%) subjects. Median follow-up of survivors was 22 months (range, 1-85) months. Erythroid and myeloid colonies were isolated from each subject with one cytogenetic abnormality such as del(5/5q), +8, del(7/7q) or del(20q). Cytogenetic abnormalities of each colony were analyzed by fluorescence in situ hybridization (FISH). SPSS 17.0 software was used to make statistical analysis.

**Results:**

The numbers of burst-forming units-erythroid (BFU-E), colony forming unit-erythroid (CFU-E) and colony forming unit-granulocytes/macrophages (CFU-G/M) were significantly lower than normals. A high ratio of cluster- to CFU-G/M was associated with poor-risk cytogenetics. In multivariable analyses a cluster- to CFU-G/M ratio >0.6 was an independent risk-factor for OS after adjusting for IPSS-R (HR 3.339, [95%CI 1.434-7.778]; *P* = 0.005) in very high-risk cohort.

**Conclusion:**

These data suggest abnormalities of proliferation and differentiation of erythroid and myeloid precursor cells *in vitro* parallel the ineffective hematopoiesis typical of MDS and may be useful in predicting outcomes of persons with higher-risk MDS.

## INTRODUCTION

Myelodysplastic syndrome (MDS) is a heterogeneous neoplasm characterized by ineffective hematopoiesis with resultant cytopenias and a substantial risk of progression to acute myeloid leukemia (AML) [1, 2]. This ineffective hematopoiesis results from several factors including: (1) abnormal function of haematopoietic progenitor cells of the neoplastic clone resulting from mutations in one or more genes controlling DNA, histone and spliceosome functions [3, 4]; and (2) suppression of the function of normal residual haematopoietic progenitor cells by the neoplastic clone [4-6]. Other mechanisms may also operate.

*In vitro* colony-forming unit cell (CFU-C) assays are usually-used to detect the quantitative and qualitative features of haematopoietic stem cells (HSCs) from normals and persons with diverse haematologic abnormalities including MDS [7]. Lower than normal frequencies of normal CFU-Cs and abnormal clusters of CFU-Cs are common in persons with MDS and were proposed as a diagnostic criterion [8-13]. We analyzed CFU-C quantity and quality in 365 consecutive, newly-diagnosed untreated subjects with MDS. Our data indicate that abnormalities of proliferation and differentiation of erythroid and myeloid precursor cells *in vitro* parallel the ineffective hematopoiesis typical of MDS and a cluster- to CFU-G/M ratio >0.6 was an independent risk-factor for overall survival after adjusting for conventional prognostic scoring systems [14].

## RESULTS

### Characteristics of CFU-Cs

Numbers of BFU-E and CFU-E in subjects with different forms of MDS were significantly lower than normals, and the ratio of cluster-G/M was higher than normals (Table 1). The numbers of CFU-G/M in subjects with refractory cytopenia with multi-lineage dysplasia (RCMD) were significantly lower than normals, but not in refractory anemia with ring sideroblasts (RARS), refractory anemia with excess blasts-1 (RAEB-1) and refractory anemia with excess blasts-2 (RAEB-2) (Table 1). Median numbers of the cluster-G/M was 10 (range, 0-82). Isolated decreases in BFU-E and/or CFU-E were detected in 158 subjects (43%). Isolated decreases in CFU-G/M were detected in 2 subjects, a decreased in BFU-E and/or CFU-E and CFU-G/M in 168 (46%), an increase in BFU-E and/or CFU-E with normal CFU-G/M in 12 (3%), an increase CFU-G/M with normal CFU-E and BFU-E in 3 subjects, an increase in BFU-E and/or CFU-E and CFU-G/M in 10 (2.7%). 12 subjects (3%) had normal numbers of CFU-Cs.

**Table 1:** CFU-Cs per 10E+5 mononuclear cells in MDS subtypes

	N	BFU-E	CFU-E	CFU-G/M	Ratio of cluster- to CFU-G/M
RA	5	**	**	**	**
RN	1	**	**	**	**
RT	2	**	**	**	**
RARS	14	18 (2-49)	34 (10-100)	16 (2-70)*	0.42 (0.17-0.80)
RCMD	209	10 (0-49)	28 (0-178)	14 (0-100)	0.38 (0-0.86)
RAEB1	70	10 (0-63)	30 (0-107)	14 (0-52)*	0.47 (0-0.94)
RAEB2	56	12 (0-54)	27 (2-99)	17 (0-49)*	0.43 (0-0.91)
MDS-U	5	**	**	**	**
Del(5q)	3	**	**	**	**

* No significant difference compared with normals.

** Too few cases to analyze.

Abbreviation: RA, refractory anemia; RT, refractory thrombocytopenia; RARS, refractory anemia with ring sideroblasts; RCMD, refractory cytopenia with multi-lineage dysplasia; RAEB-1, refractory anemia with excess blasts-1 (RAEB-1); RAEB-2, refractory anemia with excess blasts-2; MDS-U, myelodysplastic syndrome-unclassified (MDS-U); Del(5q), MDS associated with isolated del(5q).

### Cytogenetic analyses of CFU-C

**Figure 1:**
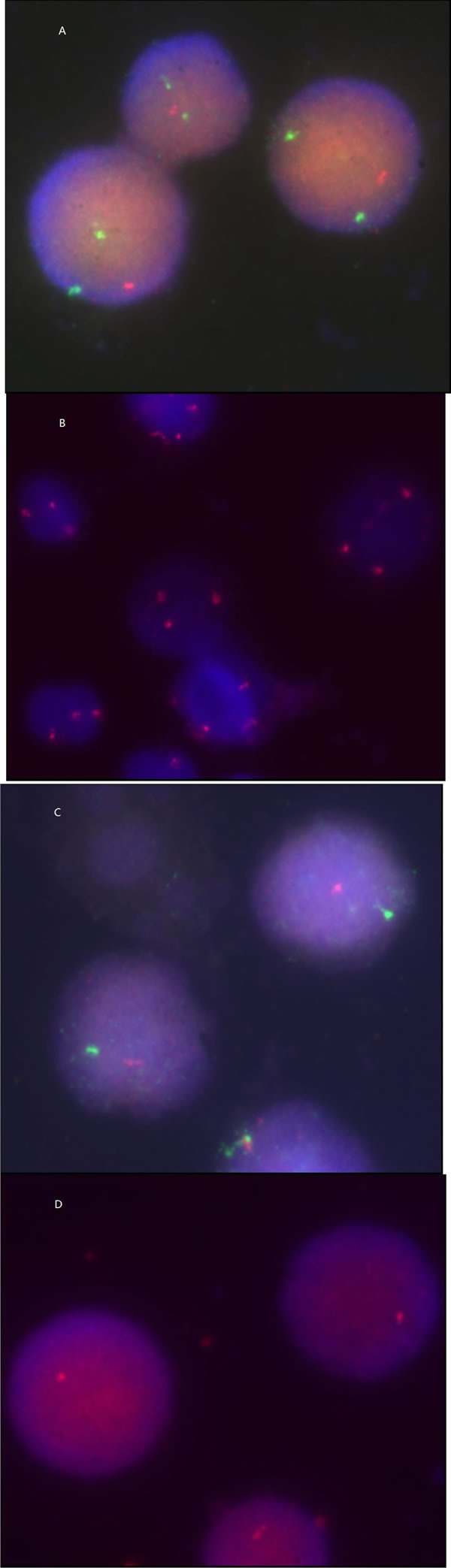
FISH of interphase nuclei in cells from colonies in subjects with del(5/5q) (A), +8 (B), -7(C) and del (20q) (D)

We used fluorescence i*n situ* hybridization (FISH) analyses of interphase nuclei to determine whether CFU-C from subjects with MDS were from the MDS clone or from residual normal progenitor cells (Figure 1). 18 subjects with del(5/5q), +8, -7 or del(20q) identified by G- and/or R-banding were studied. Abnormalities identified by FISH and by G- and/or R-banding are listed in Table 2. Sometimes cells isolated from the BFU-E were insufficient for FISH analysis. We analyzed both BFU-E and CFU-G/M from 8 subjects and only CFU-G/M from 10 subjects. Both normal and abnormal CFU-Cs were identified in 13 of 18 subjects studied.

**Table 2:** FISH analyses of cells from colonies

Subject	Karyotype	N abnormal colonies/N normal colonies	
		Myeloid	Erythroid
1	46,XY, del (5)(q13q33) [3]/ 46, XY[10]	3/2	0/4
2	47,XY,+8[4]/46,XY[16]	0/5	0/3
3	47,XY,+8[10]	0/6	2/1
4	47,XX,+8[17]	5/4	0
5	47,XX,+8[20]	9/0	0
6	47,XY,+8[10]/46,XY[6]	8/2	0
7	47,XY,+8[17]/46,XY[3]	3/2	2/2
8	47,XY,+8[3]/46,XY[17]	5/2	1/1
9	47,XX,+8[20]	6/2	0
10	47,XX,+8[20]	0/8	0
11	47,XX,+8[20]	7/4	0
12	46,XX, del (5)(q15q33)[8]	6/4	0
13	45,XY,-7 [20]	9/0	0
14	47,XX,+8[13]/46,xx[7]	2/5	0/2
15	46,XY, del(20)(q12)[16]/46,XY [4]	2/4	2/2
16	47,XY,+8[12]/46,XY[8]	5/1	1/3
17	47,XY,+8[7]	0/8	0
18	46,XY,del (5)(q13q33) [20]	9/2	0

### Associations between CFU-C and clinical and laboratory variables

Numbers of BFU-E and CFU-E were significantly associated with hemoglobin concentration and platelet level (CFU-E; *P* = 0.085 and *P* = 0.026; BFU-E; *P* < 0.001 and *P* = 0.002). Numbers of CFU-G/M were significantly associated with neutrophil level (*P* = 0.032). Subjects with hemoglobin concentrations < 100g/L (*P* = 0.001) or platelets <30×10E+9/L (*P* = 0.006) had significantly fewer BFU-E. Ratio of cluster- to CFU-G/M was not significantly-associated with percent bone marrow blasts. Subjects with higher-risk IPSS-R (*P* = 0.04) or more complex chromosome abnormalities (*P* = 0.039) were more likely to have a higher ratio of cluster- to CFU-G/M than those in the lower risk cohort or those with non-complex chromosome abnormalities (Table 3).

**Table 3:** Associations between CFU-C and clinical and laboratory variables

	*N*	BFU-E	*P*	CFU-E	*P*	CFU-G/M	*P*	Ratio of cluster- to CFU-GM	*P*
Haemoglobin (g/L)			0.001		0.162		0.587		0.024
<100	312	10 (0-63)		30 (0-178)		14 (0-100)		0.42 (0-0.94)	
≥100	53	19 (0-58)		44 (1-161)		16 (0-45)		0.33 (0-0.81)	
Platelets (x10E+9/L)			0.006		0.049		0.910		0.603
<30	100	9 (0-58)		21 (0-178)		15 (0-50)		0.40 (0-0.91)	
≥30	265	12 (0-63)		30 (0-142)		14 (0-100)		0.40 (0-0.94)	
Granulocytes (x10E+9/L)			0.674		0.269		0.396		0.725
<1.5	247	10 (0-63)		29 (0-178)		14 (0-52)		0.40 (0-0.94)	
≥1.5	118	12 (0-58)		30 (0-161)		15 (0-100)		0.40 (0-0.86)	
Bone marrow blasts (%)			0.484		0.400		0.085		0.615
<10	317	11 (0-63)		30 (0-178)		14 (0-100)		0.40 (0-0.94)	
≥10	48	11 (0-54)		32 (2-99)		18 (0-49)		0.40 (0-0.91)	
Cytogenetics (IPSS-R)			0.715		0.269		0.276		0.040
Very good	4	10 (3-18)		38 (8-48)		7 (1-28)		0.61 (0.43-0.83)	
Good	199	12 (0-49)		35 (0-178)		16 (0-70)		0.37 (0-0.8)	
Intermediate	95	10 (0-58)		29 (1-161)		14 (0-100)		0.43 (0-0.94)	
Poor	12	11 (1-34)		21 (0-98)		14 (2-40)		0.41 (0.15-0.67)	
Very poor	26	11 (0-54)		24 (2-94)		14 (0-36)		0.50 (0-0.72)	
Cytogenetic abormalities			0.822		0.144		0.296		0.039
None	182	12 (0-49)		35 (0-142)		15 (0-70)		0.35 (0-0.83)	
1	97	10 (0-58)		30 (1-178)		14 (0-100)		0.44 (0-0.94)	
2	22	10 (1-26)		29 (2-100)		16 (1-41)		0.45 (0-0.83)	
≥3	35	12 (0-54)		21 (0-98)		14 (0-40)		0.50 (0-0.72)	
IPSS-R			0.076		0.233		0.588		0.081
Very low	9	13 (3-33)		20 (4-120)		13 (4-33)		0.38 (0.10-0.71)	
Low	89	16 (0-49)		35 (0-178)		15 (0-70)		0.36 (0-0.83)	
Intermediate	118	11 (0-58)		30 (0-161)		14 (0-100)		0.38 (0-0.83)	
High	70	9 (0-49)		24 (0-103)		14 (0-49)		0.44 (0.20-0.94)	
Very high	50	10 (0-54)		25 (2-98)		16 (0-49)		0.47 (0-0.83)	

### Impact of the ratio of cluster- to CFU-G/M on outcomes

**Figure 2:**
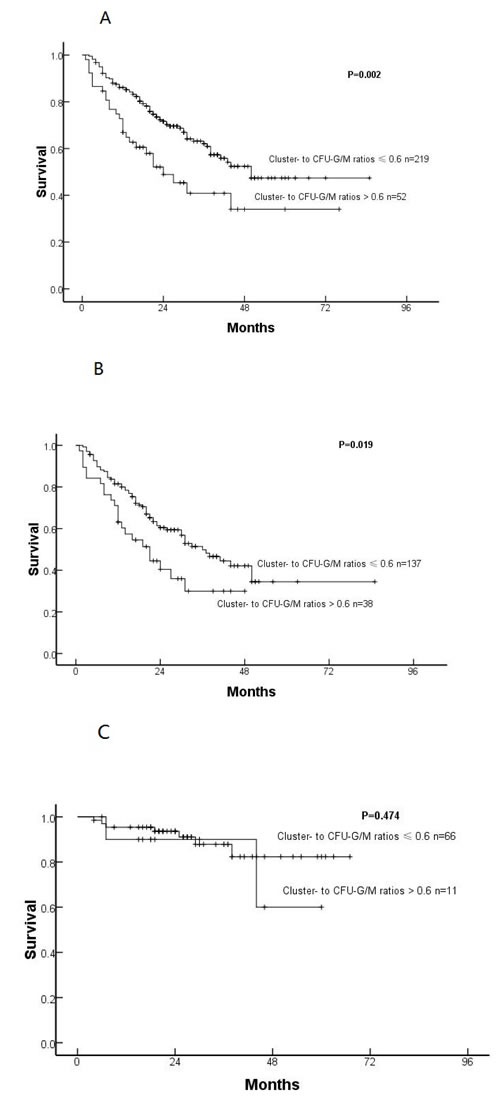
Overall survival in all subjects (A), IPSS-R higher-risk cohort (B) and IPSS-R lower-risk cohort (C) with cluster- to CFU-G/M ratios ≤or > 0.6

Subjects with a >0.6 ratio of cluster- to CFU-G/M were more likely to have briefer OS (*P* = 0.002, Figure 2A) than subjects with a ratio ≤0.6 in univariate analyses. In IPSS-R higher-risk cohort, subjects with a >0.6 ratio of cluster- to CFU-G/M were more likely to have briefer OS (*P* = 0.019, Figure 2B), but in IPSS-R lower-risk cohort, there was equal OS between subjects with a >0.6 and ≤0.6 ratio of cluster- to CFU-G/M (*P* = 0.474, Figure 2C). In multivariable analyses a cluster- to CFU-G/M ratio >0.6 was an independent risk-factor for OS after adjusting for IPSS-R (HR 3.339, [95%CI 1.434-7.778]; *P* = 0.005) in very high-risk cohort.

## DISCUSSION

In this study, we found frequent abnormalities in CFU-C in newly-diagnosed, untreated subjects with MDS. About 90 percent had reduced or absent erythroid and/or myeloid CFU-C and about 95 percent had an increased ratio of cluster- to CFU-G/M. Although in MDS patients there were significant alterations in both the myeloid and the erythroid lineages, the quantitative deficiencies observed in the erythroid lineage were more pronounced than the deficiencies found in the myeloid lineage. We also found a significant association between BFU-E and platelet levels. Our data are consistent with some previous studies CFU-C in persons with MDS [8-12].

The CFU-C assays are considered as a direct method to detect the quantitative and qualitative abnormalities of HSCs from patients with MDS. We found that the number and category of cytopenias was in parallel with CFU-C. Using FISH we determined CFU-C were often a combination of cells from the MDS clone and residual normal cells mimicking the ineffective hematopoiesis typical of MDS. We confirmed that the abnormalities of CFU-C assays in MDS reflected the existence and degree of the ineffective hematopoiesis. Therefore, our data support that CFU-C assays should be an important co-criteria for diagnosis of MDS [13].

A high ratio of cluster- to CFU-G/M was associated with poor-risk cytogenetics and OS. Multivariable analyses confirmed these associations were independent of IPSS-R Score for MDS patients, especially in very high-risk group. Therefore, we considered that the ratio of cluster-to CFU-G/M represented the malignant colonies and was associated with survival in MDS patients.

At present, it was undiscovered that what molecular mechanism drove the ineffective hematopoiesis. In a prior study we reported ASXL1 mutations were associated with relatively more colony formation of BFU-E, CFU-E and CFU-GM [15]. These data raise the possibility one or more mutations may operate in the quantitative and qualitative abnormalities of CFU-C. We are using exomic- and whole genome sequencing in our MDS patients to explore this possibility and provide some new insights into the biology of MDS.

In conclusion, we suggest CFU-C assays in persons with newly-diagnosed, untreated MDS may provide additional useful prognostic data to the IPSS-R systems. These assays may also be useful in studying biological aspects of MDS.

## MATERIALS AND METHODS

### Patient data

365 consecutive newly-diagnosed, untreated subjects with MDS diagnosed from July, 2007 to April, 2014 were studied. The study was approved by the Ethical Committee of the Institute of Hematology and Blood Diseases Hospital, Chinese Academy of Medical Science according to the guidelines of the Declaration of Helsinki and all subjects gave written informed consent. Median age was 48 years (range, 12-83 years). 240 (66%) were male. Cases were classified according to the 2008 WHO criteria [16]. 5 subjects (1%) had refractory anemia (RA), 1, refractory neutropenia (RN), 2, refractory thrombocytopenia (RT), 14 (4%), refractory anemia with ring sideroblasts (RARS), 209 (57%), refractory cytopenia with multi-lineage dysplasia (RCMD), 70 (19%), refractory anemia with excess blasts-1 (RAEB-1), 56 (15%), refractory anemia with excess blasts-2 (RAEB-2), 5 (1%), myelodysplastic syndrome-unclassified (MDS-U) and 3, MDS associated with isolated del(5q). 336 (92%) subjects with evaluable cytogenetics were classified using the revised International Prognostic Scoring System (IPSS-R) criteria. In the IPSS-R classification we combined very low- and low-risk subjects into a lower-risk cohort and intermediate-, high- and very high-risk groups into a higher-risk cohort.

Subjects were observed for survival until July 31, 2015. Follow-up data were available for 289 (80%)subjects. Median follow-up of survivors was 22 months (range, 1-85) months. 142 (39%) subjects received immune suppressive drugs including cyclosporine and thalidomide. 46 subjects (13%) received anti-cancer therapy(ies) including aclacinomycin or homoharringtonine combined with cytarabine and granulocyte-colony stimulating factor (G-CSF; termed CAG or HAG), idarubicin or daunorubicin combined with cytarabine (IA or DA) or melphalan. 75 (21%) subjects received erythropoietin with or without G-CSF, RBC and/or platelet transfusions and/or iron chelation with desferrioxamine. 11 (3%) subjects received decitabine, 19 (5%) an allotransplant and 72 (20%), traditional Chinese medicines. Subjects receiving an allotransplants were censored in survival analyses.

### CFU-C assays

**Figure 3:**
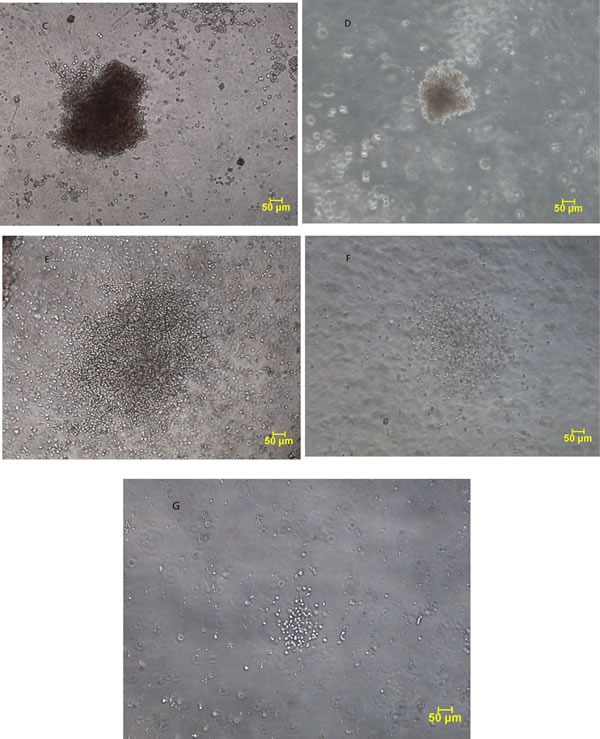
Representative examples of colonies (100×) from the normal subjects and patients with myelodysplastic syndrome Colony forming unit-erythroid (CFU-E) from the normal subject **A.** and the patient **B.** Burst-forming unit-erythroid (BFU-E) from the normal subject **C.** and the patient **D.** Colony forming unit-granulocyte/macrophage (CFU-G/M) from the normal subject **E.** and the patient **F.** Cluster-G/M **G.** from the patient.

5-10 mL of bone marrow was collected in sterile, preservative-free heparin tubes and mononuclear cells were separated by Ficoll-Hypaque gradient density centrifugation. CFU-C assays were performed using the MethoCultTM H4435 Enriched (STEMCELL Technologies, Vancouver, Canada.). Briefly, cells were plated at 1×10E+5 cells/mL into 2 wells (1 ml/well) of 24-wells plates and placed into an incubator maintained at 37°C with 5% CO2 and >85% humidity. A colony was defined as an aggregate of >40 cells. Clusters consisted of 4 to 40 cells. Colony forming unit-erythroid (CFU-E) (Figure 3A and 3B) were enumerated after 7 days and the burst-forming unit-erythroid (BFU-E) (Figure 3C and 3D) after 14 days using an inverted phase microscope. Colony forming unit-granulocyte/macrophage (CFU-G/M) (Figure 3E and 3F) and cluster-G/M (Figure 3G) were enumerated after 14 days. Colony numbers were reported as an average of 2 wells adjusted to 10E+5 mononuclear cells plated. The ratio of cluster-G/M was calculated as numbers of cluster-G/Ms divided by number of CFU-G/M and cluster-G/M. Bone marrow samples of a total of 20 healthy volunteer donors ranged from 18 to 50 yrs old were used to establish the normal reference of CFU-Cs. The reference of normal averages (Mean±SD) at our lab were: BFU-E 31±6, CFU-E 81±12, CFU-G/M 22±7 and the ratio of cluster-G/M 0.1±0.05.

### FISH studies

Cells were isolated from individual BFU-E and CFU-G/M after 14 days of culture and centrifuged onto microscopic slides. ≥5 erythroid and myeloid colonies were isolated from each subject with one cytogenetic abnormality such as del(5/5q), +8, del(7/7q), or del (20q). ≥8 colonies were analyzed by FISH using DNA probes to detect del(5/5q), +8, del(7/7q) and del(20q): 5p (D5S721), 5q (EGR1), CEP8, CEP7, D7S486 and D20S108 (Abbott Molecular, Illinois, U.S.A.). Probe sensitivities were D5S721, 3%, EGR1, 3%, CEP8, 2.5%, CEP7, 2.5%, D7S486 3%, D20S108, 2%. At least 100 interphase nuclei per colony were analyzed. Hybridization and analysis were performed by standard techniques.

### Statistics

The Mann-Whitney U-test or Kruskal-Wallis analysis of variance were used for continuous variables. Survival distributions were estimated by the Kaplan-Meier method. Cox proportional hazards regression model was used to assess the correlation between variables and survival. Overall survival (OS) was defined as time from diagnosis to date of death or last seen. Two-tailed P-values ≤0.05 were considered significant.
